# Exploring Serum Biomarker Levels in Tetralogy of Fallot, Hypoplastic Left Heart Syndrome, and Healthy Children

**DOI:** 10.1007/s00246-025-03935-0

**Published:** 2025-07-08

**Authors:** Ariel Vargas, Jose Galan, Kriyana Reddy, Angeli Thomas, Nkecha Hughes, Grace DeCost, Anh D. Mai, Andrea L. Jones, Monique M. Gardner, Laura Mercer-Rosa

**Affiliations:** 1 Division of Cardiology, Children’s Hospital of Philadelphia, Philadelphia, PA USA; 2 Atrium Health Levine Children’s Hospital, Charlotte, NC USA; 3 Department of Pediatrics, Perelman School of Medicine, University of Pennsylvania, Philadelphia, PA USA; 4 Philadelphia College of Osteopathic Medicine, Philadelphia, PA USA; 5 Dana-Farber Cancer Institute, New York City, NY USA

**Keywords:** Congenital heart disease, Tetralogy of Fallot, Biomarkers, Cardiac remodeling

## Abstract

Serum biomarkers have emerged as tools for diagnosis and management in adult heart disease but are less investigated in the pediatric population. This exploratory study reports biomarker profiles in unrepaired tetralogy of Fallot (TOF), repaired TOF (rTOF), hypoplastic left heart syndrome (HLHS) following Fontan surgery, and healthy controls. We compared circulating biomarker patterns between TOF, rTOF, HLHS, and control groups, aiming to characterize potential disease-specific profiles and generate hypotheses for future research. We prospectively enrolled subjects and collected single-time blood samples for analysis. We measured: microRNA-21 (miR-21), soluble suppression of tumorigenicity-2 (sST-2), galectin-3 (Gal-3), procollagen type-I carboxy-terminal pro-peptide (PICP), procollagen type-III amino-terminal pro-peptide (PIIINP), metalloproteinases (MMP-1/MMP-9), and NT-proBNP. We included 207 patients: TOF (*n* = 75), rTOF (*n* = 60), HLHS (*n* = 11), and healthy controls (*n* = 60). Compared to the younger controls, TOF patients had higher PICP, MMP-1, and NT-proBNP. Compared to older controls, rTOF patients had higher PIIINP, MMP-1, MMP-9, and NT-proBNP; and HLHS patients had higher PIIINP and MMP-1. Collagen metabolism biomarkers and MMP-1 were elevated across disease groups. Gal-3 was associated with age in HLHS. No disease-specific patterns were observed; however, differences from controls suggest cardiac remodeling in TOF and HLHS.

**Supplementary Information:**

The online version contains supplementary material available at 10.1007/s00246-025-03935-0.

## Introduction

In congenital heart disease (CHD), cardiac surgery is not curative, and patients experience residual lesions and ongoing morbidity [1]. Surgical repair of some CHDs can alter hemodynamics, leading to residual volume and/or pressure overload, myocardial ischemia risk, and neurohormonal activation [2]. Alongside alterations in volume and pressure loading, the myocardium undergoes adaptive remodeling to maintain systolic function and adequate filling pressures [3]. Myocardial remodeling induces changes in contractility patterns and increases risk of dysfunction [4]. Myocardial injury, inflammation, and fibrosis prompt the release of various proteins and regulators, including microRNAs, inflammatory, and growth factors, which circulate in the peripheral blood [2, 5].

Despite extensive research in adults, circulating biomarkers are understudied in children with CHD. We have previously demonstrated that MMP-1 could be a possible biomarker of ventricular remodeling after tetralogy of Fallot repair [6]. In this study, we measured a panel of biomarkers in patients with tetralogy of Fallot (TOF) before surgical repair, in patients with TOF years after repair and those with hypoplastic left heart syndrome (HLHS) presenting for clinically indicated cardiac magnetic resonance imaging, and in healthy controls. These biomarkers are involved in pathways related to cardiac remodeling, with clinical relevance in other disease states [7]. We hypothesized that serum cardiac biomarkers would be elevated in patients with TOF, rTOF, and HLHS compared to healthy children, and that disease-specific patterns reflecting cardiac remodeling would be identified, establishing a basis for further research.

## Methods

This study protocol was approved by the Institutional Review Board for the Protection of Human Subjects at Children’s Hospital of Philadelphia (CHOP), and parental informed consent, and subject assent were obtained prior to study inclusion. We conducted a single-center study with prospective enrollment of patients with TOF, HLHS, and healthy children that were followed at CHOP.

For the CHD groups, patients were eligible for inclusion if they had a diagnosis of TOF or HLHS. TOF patients were included if they presented for complete surgical repair, regardless of pulmonary valve anatomy or prior palliative procedures. For the repaired TOF (rTOF) subgroup, patients were included if they had undergone complete repair at ≤ 2 years of age, irrespective of history of prior palliation or pulmonary valve anatomy. HLHS patients were included if they were post-Fontan procedure and had previously undergone stage II palliation. All ages were included in the study to ensure broad representativity.

We obtained blood samples from unrepaired TOF infants, rTOF children and adolescents (rTOF), HLHS patients’ post-Fontan surgery, and controls. TOF patients were enrolled as part of our prospective studies focused on outcomes in TOF. HLHS patients were enrolled to serve as a comparison group with a very distinct anatomy and physiology. Healthy controls were enrolled for comparison against children with heart disease. TOF samples were collected during surgical repair (prior to cardiopulmonary bypass). rTOF and HLHS samples were obtained during standard-of-care cardiac magnetic resonance imaging (CMR) at routine follow-up visits scheduled at varying intervals postoperatively, for which a venipuncture for the purposes of research was not necessary as patients had an intravenous line in place as part of the CMR. TOF and rTOF patient groups have been previously described [6, 8–10]. Control samples were collected from a previously placed intravenous line during minor pediatric non-cardiac procedures in similar-ages healthy children without chronic illnesses or cardiac disease. Laboratory staff were blinded to sample group assignment.

### Biomarker Technique

In total, 7 biomarkers were measured based on existing literature in children and adults with congenital heart defects. Markers of cell proliferation and fibroblast function [microRNA-21 (miR-21)] [11]; inflammation and fibrosis [soluble ST-2 (sST-2) (also known as Interleukin-1 receptor-like 1) and galectin-3 (Gal-3), respectively] [7, 12–15]; collagen metabolism [procollagen type-I carboxy-terminal pro-peptide (PICP) and procollagen type-III amino-terminal pro-peptide (PIIINP)], extracellular matrix (ECM) turnover and myocardial structural modification [metalloproteinases 1 and 9 (MMP-1 and MMP-9)] [2, 6, 7, 12]; and myocyte stretch (NT-proBNP) [16, 17]. We were unable to obtain samples for miR-21 and sST-2 measurements in healthy children due to challenges in sample processing and analysis; thus, they were not included in the analytical models.

Serum and EDTA plasma samples were processed and stored at − 80 °C prior to analysis by the Translational Core Laboratory at CHOP. NT-proBNP, Gal-3, sST-2, MMP-1, and MMP-9 were quantified using validated immunoassays (ELLA and MSD platforms), with all measurements performed in replicate by personnel blinded to clinical data. Inter- and intra-assay variability were within acceptable limits. Total RNA was extracted from serum using the Applied Biosystems miRNA Serum Kit. Quantitative RT-PCR for miR-21 was performed using commercially available primers, normalized to miR-16, and analyzed via the 2^−ΔΔ*Ct*^ method. Melt curve analysis confirmed amplification specificity. Specific details on the methodology, technique, and processes used to analyze each biomarker are provided in the supplementary materials [18] (Online Resource 1).

### Statistical Analysis

Continuous variables are described using mean with standard deviation (SD) or median with interquartile range (IQR), if not normally distributed. Categorical variables are described with counts and percentages. Biomarker comparisons between groups were performed using Wilcoxon rank-sum tests, as the data were non-normally distributed across all groups. To approximate age matching with the CHD groups, healthy controls were stratified into two groups using a ≥ 2-year age cutoff for Wilcoxon rank-sum tests. This stratification was employed to address the practical limitations of enrolling precisely age-matched healthy pediatric controls.

Spearman correlation coefficients assessed the association between biomarker levels and age within each group, with coefficients ≤ 0.3 as modest, 0.3–0.7 as moderate, and ≥ 0.7 as strong. Statistical significance was set at *p* < 0.05. For this analysis, all control subjects were considered as a single group and not subdivided. Data analysis was conducted using STATA 14.1 (Stata Corp, College Station, TX).

## Results

### Clinical Characteristics

A total of 205 patients with serum biomarker measurements were included, divided into four groups: TOF (*n* = 75), rTOF (*n* = 60), HLHS (*n* = 11), and controls (*n* = 59).

For TOF, most patients were male [*n* = 46 (61.3%)] and White [*n* = 56 (74%)]. The median age at sampling was 3.72 months (IQR 2.64, 5.52). The rTOF group had more females [*n* = 33 (55%)] and White individuals [*n* = 51 (85%)], with a median age at sampling of 15 years (IQR 9, 22). HLHS patients included 7 (64%) females and mostly White individuals [*n* = 10 (91%)]. The median age at sampling was 10.1 years (IQR 2.59, 12.9). For controls, the median age at sampling was 1.37 years (IQR 1.05, 1.69) in the < 2-year group (*n* = 44) and 3.27 years (IQR 2.93, 6.66) in the ≥ 2-year group (*n* = 16) (Table 1).

**Table 1:** Demographic characteristics and biomarker levels per group

	TOF (*n* = 75)	Controls < 2 y/o (*n* = 44)	rTOF (*n* = 60)	HLHS (*n* = 11)	Controls ≥ 2 y/o (*n* = 16)
Male *n* (%)	46 (61.3)	30 (68%)	27 (45)	4 (36)	7 (44)
White race *n* (%)	56 (74)	NA	51 (85)	10 (91)	NA
Age at sampling, years	0.31 (0.22, 0.46)	1.37 (1.05, 1.69)	15 (9, 22)	10.1 (2.59, 12.9)	3.27 (2.93, 6.66)

Biomarker (ng/mL)		*p*-value#			*p*-value#		*p*-value#	
miR-21	27.79 (26.52, 30.77)		NA	28.1 (26.9, 29.05)		27.63 (26.5, 30.49)		NA
sST-2	13.4 (9.15, 20.5)		NA	15.05 (12.05, 19.75)		17.07 (14.85, 24.16)		NA
Gal-3	3.75 (3.17, 4.56)	** < 0.001**	6.82 (5.41, 32.58)	5.44 (4.6, 6.36)	**0.007**	5.09 (4.54, 5.98)	**0.0057**	6.7 (5.88, 7.93)
PICP	6400 (6146.6, 6400)	** < 0.001**	3580.3 (2784.1, 4925.40)	1192.3 (822.3, 1782.9)	** < 0.001**	1055.9 (923.35, 3591.7)	0.32	2471.25 (2114.10, 3118.20)
PIIINP	11.3 (5.6, 20)	0.36	12.05 (9.40, 18.45)	20 (16.5, 20)	** < 0.001**	20.00 (15.7, 20)	**0.006**	10.65 (8, 19.10)
MMP-1	11.6 (5.09, 18.39)	**0.04**	6.71 (4.85, 11.01)	14.62 (10.29, 26.34)	** < 0.001**	18.11 (9.84, 36.2)	**0.006**	6.44 (5.68, 10.92)
MMP-9	65.73 (46.9, 91.9)	** < 0.001**	136.97 (82.96, 205.72)	133.72 (81.08, 236.5)	**0.01**	127.23 (96.65,181.04)	0.19	75.44 (67.35, 115.48)
NT-proBNP	0.67 (0.3, 1.6)	** < 0.001**	0.10 (0.06, 0.20)	0.29 (0.1, 0.73)	**0.02**	0.51 (0.11, 1.3)	0.10	0.12 (0.10, 0.22)

Bold numbers indicate a significant P-value of < 0.05

*TOF* unrepaired TOF infants, *rTOF* repaired TOF children and adolescents, *HLHS* Hypoplastic Left Heart Syndrome post-Fontan surgery

*Values reported as median, (IQR)

^#^Wilcoxon rank-sum tests *p*-values for biomarker comparison between TOF, rTOF, and HLHS with controls

### Biomarker Measurements

The measurements of biomarkers for all groups for comparison are reported in Table 1. Fifty-six (75%) TOF infants and eight (13%) controls presented PICP levels exceeding the maximum level of detection (6400 ng/mL). Twenty-one (28%) TOF infants and nine (75%) HLHS patients had PIIINP levels above the detection limit (20 ng/mL). Table 1 shows miR-21 and sST-2 values in CHD patients.

Compared to controls, TOF patients had higher levels of PICP (*p* < 0.001), MMP-1 (*p* = 0.04), and NT-proBNP (*p* < 0.001), and lower levels of MMP-9 and Gal-3 (*p* < 0.001 for both). PIIINP levels were similar and not significantly different (*p* = 0.36).

Compared to controls, rTOF had higher levels of PIIINP (*p* < 0.001), MMP-1 (*p* < 0.001), MMP-9 (*p* = 0.01), NT-proBNP (*p* = 0.02), and lower levels of PICP (*p* < 0.001) and Gal-3 (*p* = 0.007).

Compared to controls, HLHS patients had higher levels of PIIINP (*p* = 0.03), and MMP-1 (*p* = 0.006), and lower levels of Gal-3 (*p* = 0.0057). There were no significant differences in PICP (*p* = 0.32), MMP-9 (*p* = 0.65), and NT-proBNP (*p* = 0.10).

No significant differences were found between biomarker values in rTOF and HLHS groups. Biomarker levels for all four groups (TOF, rTOF, HLHS, and controls), as well as comparisons with controls, are presented in Table 1.

### Association Between Age and Biomarker Levels

Moderate negative associations were observed between age and PICP levels in TOF (*ρ* = − 0.51, *p* < 0.001), rTOF (*ρ* = − 0.63, *p* < 0.001), and controls (*ρ* =  − 0.61, *p* < 0.001), with younger patients having higher PICP levels. A strong negative association was found between age and miR-21 levels in HLHS (*ρ* = − 0.74, *p* = 0.005). There was a strong direct association between age and Gal-3 levels in HLHS (*ρ* = 0.81, *p* = 0.001). rTOF patients showed a modest positive association between age and sST-2 (*ρ* = 0.30, *p* = 0.02) and a moderate positive association between age and MMP-9 (*ρ* = 0.44, *p* < 0.001) levels (Table 2).

**Table 2:**
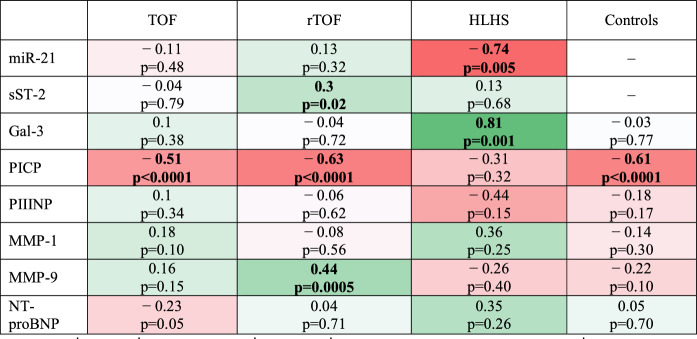
Correlation heat map between biomarker levels and age per group

Bold numbers indicate a significant P-value of < 0.05

*TOF* unrepaired TOF infants, *rTOF* repaired TOF children and adolescents, *HLHS* Hypoplastic Left Heart Syndrome post-Fontan surgery. Red indicates negative correlations and green represents positive correlations. Statistical significance was stablished at *p* < 0.05

## Discussion

This exploratory study provides a descriptive report of serum cardiac biomarker profiles in a group-specific manner in patients with TOF, rTOF, HLHS, and controls, comparing these findings between groups. We present associations between age and biomarker levels, especially for fibrosis-related biomarkers.

miR-21 levels are reported for the CHD groups in our study. Previous studies in patients with diverse cardiac disease states, including rTOF, dilated cardiomyopathy (DCM), pulmonary hypertension, systemic hypertension, and heart failure (HF) have reported elevations or increased expression of this biomarker [6, 19–23]. miR-21 acts as a regulator and non-specific marker of cardiac hypertrophy and fibrosis, as demonstrated in adult populations and animal models with left ventricular (LV) dysfunction [11, 19, 24–29], and is highly expressed in cardiac fibroblasts, targeting genes associated with RV pressure overload and the transition from hypertrophy to HF [20, 24, 30]. However, the molecular events driving the progression from compensated states to HF in CHD, where the RV is primarily affected, remain unclear. While data on miR-21 references values in the general population are limited, we theorize that miR-21 could be upregulated in CHD, and this may reflect an active cardiac remodeling process.

sST-2 reference ranges have been proposed in healthy pediatric populations, with proposed references values of 2.1-21.0 ng/mL, 9-50 ng/mL, and 2.4-26.4 ng/mL [31–33]. sST-2 elevation is associated with adverse outcomes in adults with HF. sST-2 is induced in mechanically overloaded myocytes through a mechanism involving Interleukin-33 and sST2’s transmembrane receptor, counteracting regulatory fibrosis and inflammation mechanisms [6, 34, 35]. We investigated sST-2 due to this mechanism and because sST-2 offers prognostic and additive risk stratification information in acute and chronic HF when combined with complementary validated biomarkers, such as NT-proBNP [34–38]; however, we were limited in our analysis of sST-2 but demonstrate a direct association with age in the rTOF patient group. We acknowledge that sST-2 diagnostic utility in HF and children with CHD remains limited, and serial measurements may offer greater value [39–41].

We found significantly lower Gal-3 levels in the TOF, rTOF, and HLHS groups compared to controls. Gal-3 is a biomarker for both cardiac and non-cardiac fibrosis by promoting myofibroblast attraction, adhesion, and proliferation, as well as myocardial collagen deposition [42–44]. It has been extensively investigated in adult HF and linked with cardiac fibrosis, early myocardial dysfunction, disease progression, and increased mortality [36, 45–50]. In Fontan adults, Gal-3 increases with age, but shows no association with specific cardiac anatomy or Fontan type [43]. Only in our very small HLHS cohort, we identified a strong positive correlation between age and Gal-3 levels, in line with prior reports demonstrating age-related increases [51].

Although absolute Gal-3 concentrations were lower than those of controls, this may reflect population-specific dynamics or methodological differences, rather than a true divergence from the established trend. Karatolios et al. [52] reported that lower levels of Gal-3 (< 59 ng/mL) are associated with LV reverse remodeling in adults with recent onset DCM. In this context, the lower Gal-3 concentrations observed in our TOF, rTOF, and HLHS groups may suggest that the myocardial remodeling process has not yet reached a stage at which Gal-3 is upregulated, reflecting an earlier or distinct phase of myocardial remodeling [53]. Gal-3 is shown to be elevated in pediatric patients with HF secondary to CHD [54, 55], non-cyanotic heart disease [56], pulmonary hypertension, and rTOF [6, 57]. Further research is needed to elucidate the lower Gal-3 levels observed in our CHD groups compared to controls, as well as the temporal and mechanistic aspects of Gal-3 regulation in CHD. These differences may be influenced by intrinsic variability in serum levels among individuals with cardiac disease, elevations in healthy individuals related to non-cardiac inflammation, and differences in group sizes [45, 53, 58, 59].

Differences in PICP levels may be attributed to the ECM being a metabolically active environment, where fibrosis markers fluctuate depending on the balance between collagen synthesis and degradation [60]. Interestingly, PICP levels were highly elevated in most TOF patients, but also in eight controls. This could be explained by the dynamic nature of collagen turnover in both healthy and disease states [61]. Prior studies in HF, DCM, and systemic hypertension have shown that elevated PICP levels at baseline are positively correlated with myocardial collagen assessed histologically and are associated with increased mortality [42, 60, 62–64]. These findings suggest that PICP levels could be directly related to myocardial collagen turnover and may offer valuable preliminary insight into cardiac remodeling in children with CHD.

Our rTOF and HLHS patients exhibited elevated PIIINP levels compared to controls. Serum PIIINP is elevated in conditions that result in myocardial fibrosis, such as CHD and DCM, and correlates with myocardial collagen type-III content [64–69]. Prior studies report PIIINP elevations before and after Fontan surgery, with levels decreasing across staged palliation surgeries, suggesting a response to reduced cyanosis and volume load [65, 70]. However, fibrogenesis may persist even after surgical improvement of ventricular overload [65], as seen in our HLHS patients. In TOF, PIIINP correlates with the degree of cyanosis [5], and higher levels have been reported in the setting of persistent hemodynamic stress from pressure and/or volume overload, as well as ongoing fibrotic remodeling despite repair, which may explain our TOF group findings [66]. While these mechanisms may help contextualize our findings in rTOF, the absence of detailed data on hemodynamic status and the use of therapies that may prevent remodeling in our cohort limit our ability to draw definitive conclusions.

Lai et al. [71] demonstrated that PICP is a more sensitive fibrosis marker than PIIINP due to the fixed 1:1 ratio of PICP molecules released after collagen type-I cleavage, whereas PIIINP and collage type-III lack a stoichiometric ratio, where the number of molecules released into the circulation varies due to incomplete cleavage of procollagen type-III [60, 72, 73]. For both, hepatic clearance should be considered, as higher levels may result from reduced hepatic function, especially in HLHS, where the liver function may be compromised due to Fontan circulation [60, 69]. The levels of serum collagen synthesis derivatives can be negatively affected by age related to normal slowing growth rate [67, 74], as reflected by the strong negative association observed for PICP (Table 2) and the lower levels found in our slightly older rTOF cohort compared to controls aged ≥ 2 years. PICP and PIIINP may be used together to determine remodeling as CHD triggers compensatory mechanisms that promote fibrosis, regardless of CHD type or anatomical variations [5, 65, 66] pending further research in pediatric CHD.

MMPs are enzymes that mediate collagen degradation in the ECM [60, 73]. Elevated MMP-1 levels are associated with RV remodeling in children with rTOF, and LV dilation and dysfunction in adults with HF and DCM [6, 73, 75, 76], while MMP-9 levels have been associated with lower EF, remodeling, and worse clinical outcome in children with HLHS [64, 77–80]. In our study, MMP-1 levels in TOF, rTOF, and HLHS groups were higher than controls, which may reflect active ECM turnover in CHD. While these findings could suggest ongoing remodeling regardless of cardiac diagnosis or surgical status, it remains speculative and warrants further investigation. The consistent elevation of MMP-1 across distinct CHD subtypes is an encouraging signal of its potential relevance as a biomarker of ongoing myocardial remodeling. MMP-9 levels were significantly lower in the TOF group compared to controls, in contrast to findings in adult cardiovascular disease, where elevated levels are typically associated with active collagen degradation [60, 77, 81, 82]. However, levels were elevated in the rTOF group, which is consistent with ongoing myocardial remodeling following surgical repair, likely driven by persistent hemodynamic alterations and extracellular matrix turnover. As collagen metabolism is a dynamic process, various interactions and inhibitors can affect the levels of MMPs at the time they are measured [75, 83]. No data exist on the performance of MMP-1 or MMP-9 as reliable fibrosis biomarkers in pediatric CHD for clinical practice [84]. While these biomarkers show potential, further research is needed to validate their utility in clinical settings. At this time, their use in clinical practice cannot be recommended until additional studies confirm their value in identifying cardiac remodeling in pediatric CHD.

NT-proBNP levels were higher in the TOF, rTOF, and HLHS groups compared to controls, although the difference did not reach statistical significance in the HLHS group. NT-proBNP is secreted by cardiomyocytes in equal concentrations to BNP in response to ventricular stretching [85] and is associated with ventricular volume overload and impaired exercise capacity in CHD [5, 86–88]. NT-proBNP levels increase as cardiac dysfunction progresses, serving as a useful complementary biomarker for the evaluation of HF in CHD [5, 85, 89]. Multiple studies comparing NT-proBNP values between pediatric CHD, HF patients, and controls have found higher serum levels in children with CHD, further elevated in more complex heart disease. While NT-proBNP decreases through stages of palliation (with volume unloading of the SV), levels remain elevated, similarly to our findings [90–98]. Although we did not observe any significant associations between age and this biomarker, age remains a critical factor in the interpretation of NT-proBNP in pediatric populations due to developmental differences in cardiac physiology and the age-dependent etiologies of cardiac disease, in contrast to adults [85, 90, 95, 99]. These findings reinforce the established role of NT-proBNP as a valuable biomarker for assessing ventricular volume overload and cardiac dysfunction in CHD. Our results align with the existing body of literature, further emphasizing the relevance of NT-proBNP in the context of CHD and its utility in monitoring disease progression.

## Limitations

This study included a small sample of patients for all CHD groups, and a small sample of controls, especially when restricting the age for more appropriate comparison; however, biomarker collection was performed specifically for the study with excellent data quality. Selection bias cannot be ruled out, as the study includes populations with TOF, rTOF, HLHS, and healthy controls who receive care at our institution and provided informed consent for participation. While this study provides valuable insight into circulating biomarker profiles in TOF, rTOF, and HLHS, it is important to acknowledge the underlying heterogeneity in pathophysiology within each group. Individual variability, including comorbidities and clinical factors, was not fully captured in our analysis and may have influenced the observed biomarker patterns. Therefore, these initial results are exploratory and may not be generalizable to the broader CHD population. Since participants were pediatric, and most studies to date have been conducted in animal models or adults, our findings cannot be directly extrapolated to adult populations. Nonetheless, these findings offer a meaningful foundation for further investigation into disease-specific mechanisms and their clinical implications.

Biomarkers were measured in serum, whereas some studies in the literature have utilized plasma. Differences in sample matrix composition and processing may affect biomarker concentration, making direct comparisons across studies challenging.

The small HLHS sample may have influenced our results; thus, the strong correlation between age and Gal-3 levels highlights the need for further research with larger sample sizes. miR-21 and sST-2 were not measured in the control group, limiting direct comparison and interpretation of our current findings. Future studies are planned to include these measurements in healthy controls to enable more comprehensive analysis. Additionally, miR-21 was selected based on our literature review and prior experience with this biomarker, so the possibility of selection bias cannot be excluded. For most biomarkers, cardiac origin cannot be confirmed, as many are novel and non-specific to cardiac pathology, and some are present in healthy states.

## Conclusion

In this study, we analyzed a panel of biomarkers and found significant differences in circulating levels between children with TOF, rTOF, HLHS, and healthy controls, representing various stages in the cardiac fibrosis cascade, which could be associated with cardiac remodeling and dysfunction in children with CHD. While no disease-specific biomarker patterns were identified, the biomarkers reported here could offer a comparison for future studies. The strong association of Gal-3 with age, along with the consistent elevation of collagen metabolism markers and MMP-1 across CHD groups, merits further investigation and underscores the importance of accounting for age in future analyses. High biomarker levels do not imply causality, and whether this represents true cardiac remodeling in TOF, rTOF, and HLHS remains to be elucidated. Our findings contribute to the growing body of literature on biomarkers in congenital heart disease and provide a foundation for future studies. Future work should explore biomarker levels throughout disease progression to better understand their role.

## Supplementary Information

Below is the link to the electronic supplementary material.

Supplementary file1 (DOCX 22 KB)

## Data Availability

No datasets were generated or analysed during the current study.

## Abbreviations

CHD: Congenital heart disease
CMR: Cardiac magnetic resonance
DCM: Dilated cardiomyopathy
Gal-3: Galectin-3
HF: Heart failure
HLHS: Hypoplastic left heart syndrome
LV: Left ventricle
miR-21: MicroRNA-21
MMP-1: Metalloproteinase-1
MMP-9: Metalloproteinase-9
PICP: Procollagen Type-I carboxy-terminal pro-peptide
PIIINP: Procollagen type-III amino-terminal pro-peptide
RV: Right ventricle
rTOF: Repaired tetralogy of Fallot
sST-2: Soluble suppression of tumorigenicity
SV: Single ventricle
TOF: Tetralogy of Fallot
