# Supplementary material for: Exploring Serum Biomarker Levels in Tetralogy of Fallot, Hypoplastic Left Heart Syndrome, and Healthy Children
Source: Pediatr Cardiol. 2025 Jul 8;47(4):1552–61. doi: 10.1007/s00246-025-03935-0 (PMC12946379; doi:10.1007/s00246-025-03935-0)
Supplement: Supplementary file 1 — Supplementary file1 (DOCX 22 KB) [file 246_2025_3935_MOESM1_ESM.docx]

Serum and EDTA plasma samples were processed and frozen at −80 ◦C for further analysis by the biomarker core laboratories from the Translational Core at CHOP (Philadelphia, PA; <https://tcl.research.chop.edu/>) for NT-proBNP, Gal-3, sST-2, MMP-1, and MMP-9. Biomarkers were assayed using commercially available reagents by personnel blinded to clinical status.

Serum total RNA was isolated with miRNAs Serum Kit (Applied Biosystems, Foster City, CA), strictly following the manufacturer’s descriptions. RNA concentrations were measured by assessing the absorbance at 260 nm. Quantitative RT-PCR was performed as described in the manual instructions. The primers of miR-21 were purchased from Applied Biosystems (assay #: 000397). miR-21 levels were normalized to miR-16 (assay #: 000391), chosen and calculated as an internal control. Additionally, melt curves were performed to analyze the specificity. The results for each serum sample were presented with the 2-ΔΔCt method as previously described [18]. Briefly, every single sample was calculated by the differences of threshold cycle (CT) between miR-21 and miR-16: ΔCT = (CTΔmiR-21–CTΔ miR-16).

sST-2 was measured using the ELLA Automated Immunoassay System (Protein Simple; San Jose, California). Samples were tested in triplicates, and the mean %CV was 7.43 (range 0.21–7.56) out of 133 samples. Inter-plate %CV was 0.60% for QC1 (254–423 pg/ml) and 1.46% for QC2 (12,679–21,131 pg/ml). Gal-3 was also measured on the ELLA system. The average %CV was 6.43 (range 0.18–9.59) out of 133 samples. Inter-plate %CV was 1.24% for QC1 (22.3–37.2 pg/ml) and 2.10% for QC2 (1195–1992 pg/ml).

Gal-3 was also measured in triplicate on the ELLA system. The average %CV was 1.68% (range 0.18–9.59%, n = 133). Inter-plate %CV was 1.24% for QC1 (mean = 26.3 pg/ml, range = 22.3–37.2 pg/ml) and 2.10% for QC2 (mean = 1322 pg/ml, range = 1195– 1992 pg/ml).

MMP-1 and MMP-9 were measured using a 2-plex kit also from MSD (Cat#: K15034C-2). Samples were tested in duplicate and the mean %CV was 3.48% for MMP-1 and 3.47% for MMP-9 (range MMP-1 = 0.17–9.80%, range MMP-9 = 0–9.84%; n = 133). Inter-plate %CV for QC1 was 8.43% for MMP-1 (mean = 89.3 pg/ml, range = 75.0–125 pg/ ml) and 9.43% for MMP-9 (mean = 508 pg/ml, range = 375–625 pg/ml); for QC2 the inter-plate %CV was 6.19% for MMP-1 (mean = 918 pg/ml, range = 750–1250 pg/ml) and 1.42% for MMP-9 (mean = 4565 pg/ml, range = 3750–6250 pg/ml); the inter-plate %CV for QC3 was 6.57% for MMP-1 (mean = 9232 pg/ml, range = 7500–12,500 pg/ml) and 6.04% for MMP-9 (mean = 41,326 pg/ml, range = 37,500–62,500 pg/ml). sST2 was measured using the ELLA Automated Immunoassay System (Protein Simple; San Jose, California). Samples were tested in triplicate, and the mean %CV was 1.91% (range = 0.21–7.56%, n = 133). Inter-plate %CV was 0.60% for QC1 (mean = 365 pg/ml, range = 254–423 pg/ml) and 1.46% for QC2 (mean = 14,285 pg/ml, range = 12,679–21,131 pg/ml).

NT-proBNP was measured using kits (Cat#: K151JKC-2) from the Meso Scale Discovery (MSD; Rockville, Maryland). Samples were tested in duplicate and the mean %CV was 5.43% (range = 0.01–9.90%, n = 138). Inter-plate %CV was 7.21% for QC1 (mean = 11.4 pg/ml, range = 7.50–12.5 pg/ml), 6.52% for QC2 (mean = 89.3 pg/ml, range = 75.0–125 pg/ml), and 4.93% for QC3 (mean = 997.5 pg/ml, range = 750.0–1250 pg/ml).
